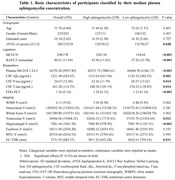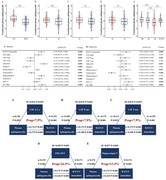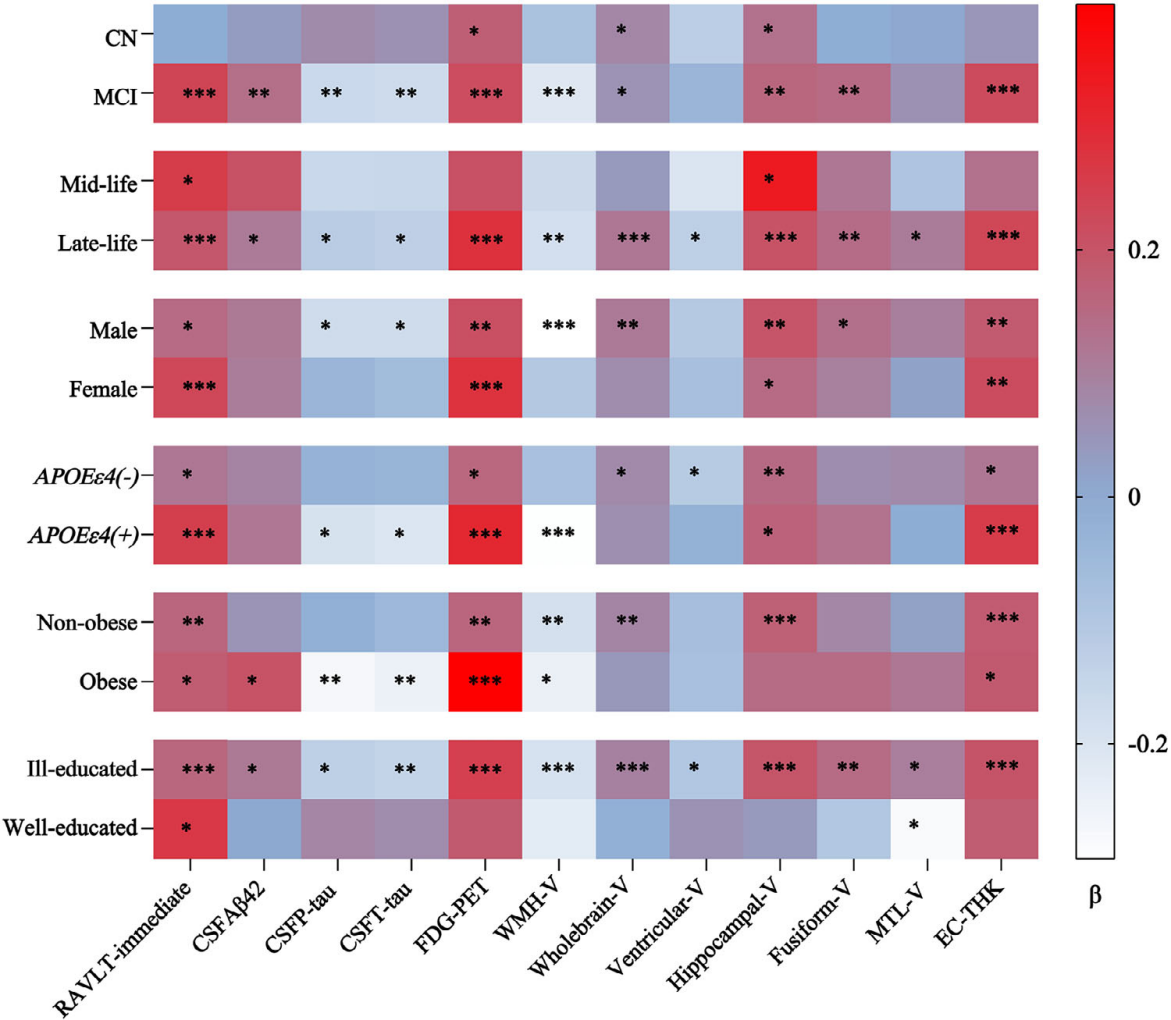# Association of sphingomyelin with Alzheimer’s pathology and cognition in non‐demented adults

**DOI:** 10.1002/alz.088121

**Published:** 2025-01-09

**Authors:** Fan Guo, Ze‐Hu Sheng, Lingzhi Ma, Ya‐nan Ou, Qing‐Fei Zhao, Tan Meng Shan, Lan Tan

**Affiliations:** ^1^ Department of Neurology, Qingdao Municipal Hospital, Qingdao university, Qingdao, Shandong China; ^2^ Department of Geriatrics, The First Affiliated Hospital of Chongqing Medical University, Chongqing Medical University, Chongqing, Chongqing China; ^3^ Department of Neurology, Qingdao Municipal Hospital, Qingdao University, Qingdao, Shandong China; ^4^ Qingdao university, Qingdao China; ^5^ Qingdao Municipal Hospital, Qingdao University, Qingdao China; ^6^ Qingdao Municipal Hospital, Qingdao university, Qingdao, Shandong China; ^7^ Qingdao Municipal hospital, Qingdao university, Qingdao, Shandong China

## Abstract

**Background:**

Plasma sphingolipids were discovered to identify memory impairment and Alzheimer’s disease (AD) risk. It has been reported to play a role in the pathological processes of neurodegeneration and neuroinflammation; however, its exact mechanism in AD has not yet been completely found.

**Method:**

A total of 476 non‐demented participants from Alzheimer’s Disease Neuroimaging Initiative were included. Multivariate cox regression was used to detect AD risk. Multiple linear regression and mixed effects modles were used to examine the sphingomyelins relationship with cerebrospinal fluid (CSF) AD biomarkers, white matter hyperintensities and cognitive. Causal mediation analysis was conducted through 10,000 bootstrapped iterations to explore the mediating effect of AD pathology on cognitive.

**Result:**

Multivariable Cox models showed that higher plasma sphingomyelin levels had lower risk of AD (HR = 0.9992; 95% CI, 0.9991 ‐ 0.9994; p < 0.004). Plasma sphingomyelin was positive correlated with RAVLT‐immediate scores, CSF Aβ42 levels, FDG‐PET, whole brain‐V, hippocampal‐V, fusiform‐V and EC‐THK. Meanwhile higher sphingomyelin is associated with lower CSF P‐tau and T‐tau levels, WMH and ventricular‐V. Higher plasma sphingomyelin can predict slower decrease of RAVLT‐immediate scores, FDG‐PET, whole brain‐V, fusiform‐V, MTL‐V, EC‐THK and increase of ventricular‐V. We found the association between sphingomyelin and verbal memory was partially mediated by levels of baseline CSF Aβ42, P‐tau and T‐tau; by FDG‐PET; and by hippocampal‐V. We found sphingomyelin interacted with cognitive diagnosis and APOEε4 status. The above results can be repeated and more pronounced in MCI, APOEε4 (+) groups.

**Conclusion:**

This study demonstrates higher plasma sphingomyelin was associated with lower AD risk and better verbal memory. Additionally, AD core pathology, along with brain metabolization and hippocampal atrophy, may mediate the association between plasma sphingomyelin and cognition. How sphingomyelin integrated within memory and AD pathways, suggests a novel possibility for identifying markers for early detection and potential avenues for effective therapeutic intervention in AD.